# Supplementary material for: Full-length transcript characterization of SF3B1 mutation in chronic lymphocytic leukemia reveals downregulation of retained introns
Source: Nat Commun. 2020 Mar 18;11:1438. doi: 10.1038/s41467-020-15171-6 (PMC7080807; doi:10.1038/s41467-020-15171-6)
Supplement: Supplementary file 1 — Supplementary Information [file 41467_2020_15171_MOESM1_ESM.pdf]

Supplementary Information

Full-length transcript characterization of *SF3B1* mutation in chronic lymphocytic leukemia reveals downregulation of retained introns

Tang et al.

Supplementary Figures

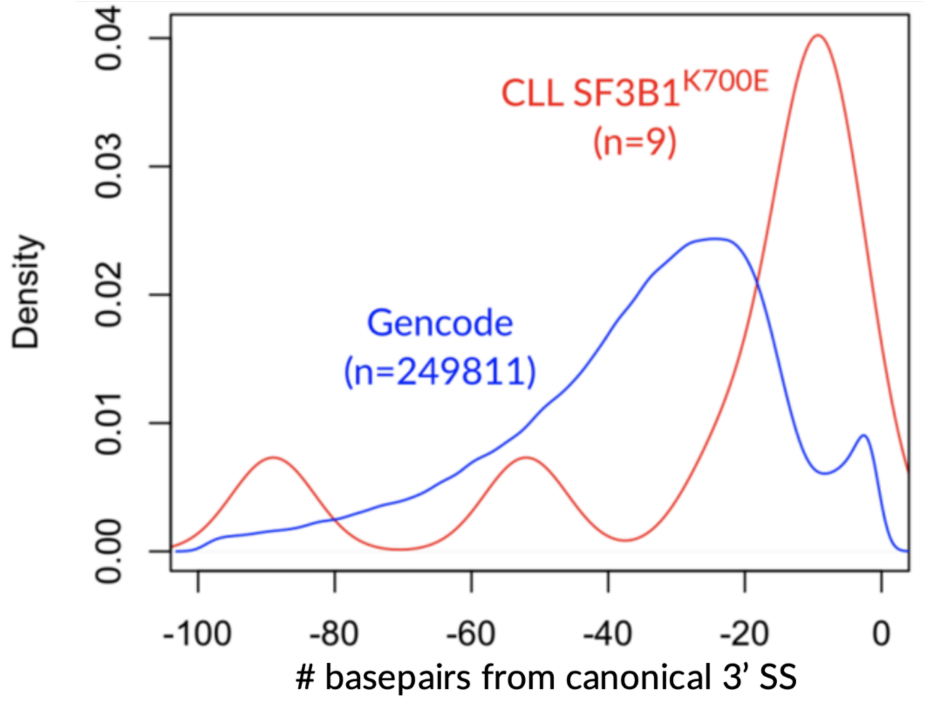

Supplementary Figure 1 | MinION alternative 3' splice site usage results. Red line, Distance between the alternative 3' SS between SF3B1^{K700E} and SF3B1^{WT} ($p < 0.1$, red line) and canonical splice sites. Blue line, the distance between canonical GENCODE v24 basic annotated 3' SS to the first non-GAG trimer (blue line).

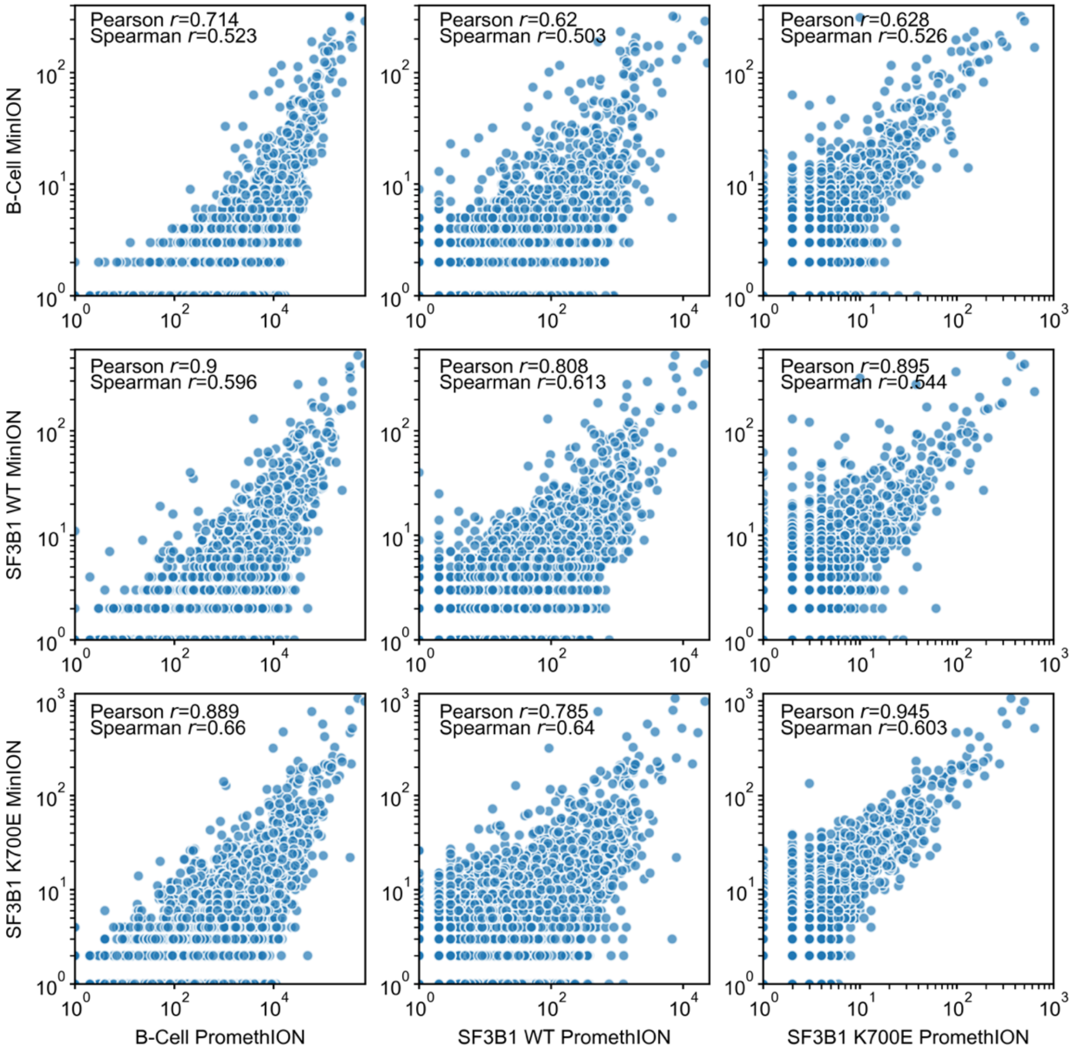

Supplementary Figure 2 | MinION and PromethION gene expression correlation. Gene expression correlation between each of the runs of samples B-cell 1, CLL SF3B1 WT 1, and CLL SF3B1 K700E 1 sequenced with both the MinION and PromethION. Gene counts were determined using primary alignments with map quality scores greater than 0 and log-scaled.

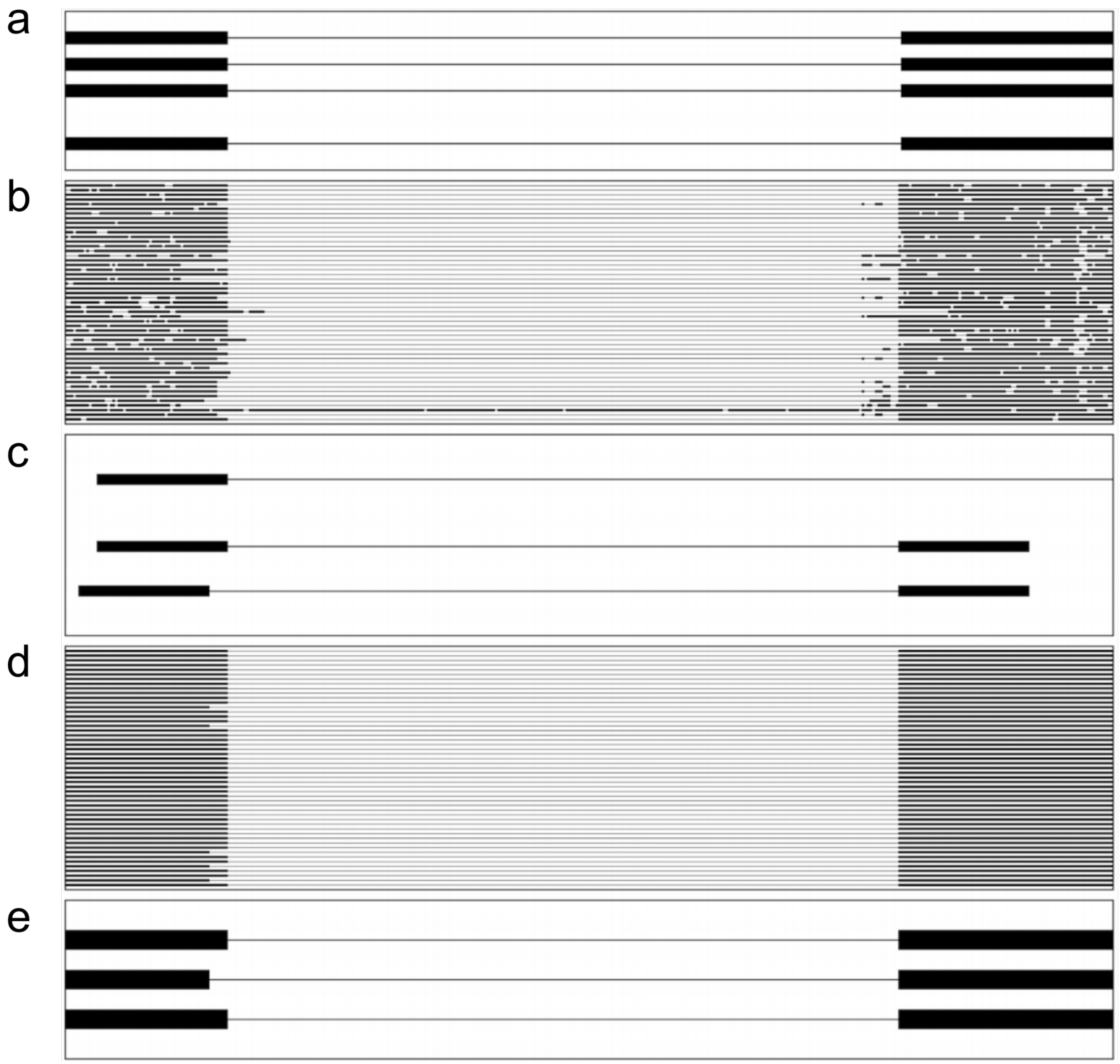

Supplementary Figure 3 | Correcting splice junctions in nanopore read alignments. a, View of a single junction for all isoforms of *FTH1* as shown in GENCODE v24 basic annotation. All following figures are of the same junction as **(a)**. **b,** A subset of the nanopore reads aligned to the genome using minimap2. **c,** Splice junctions observed in matched short read data. **d,** Splice- and gap-corrected nanopore reads using splice junctions from (**a**) and (**c**). **e,** First-pass isoform assembly by collapsing reads with the same splice junctions.

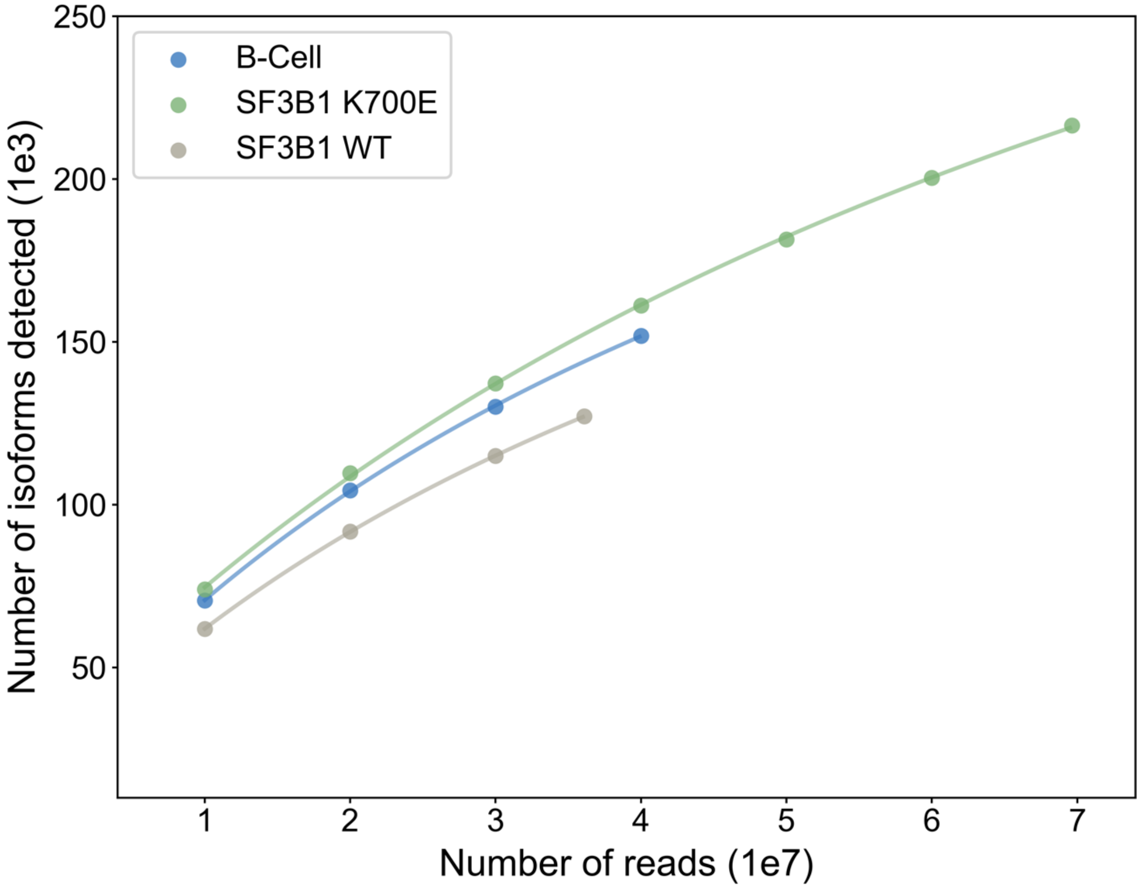

Supplementary Figure 4 | FLAIR isoform saturation by condition. Saturation plot of the number of isoforms that FLAIR identified for each subsampled set of nanopore reads. Reads were subsampled from one run from each of the conditions in increments of 10 million reads, terminating with the total number of reads for that sample.

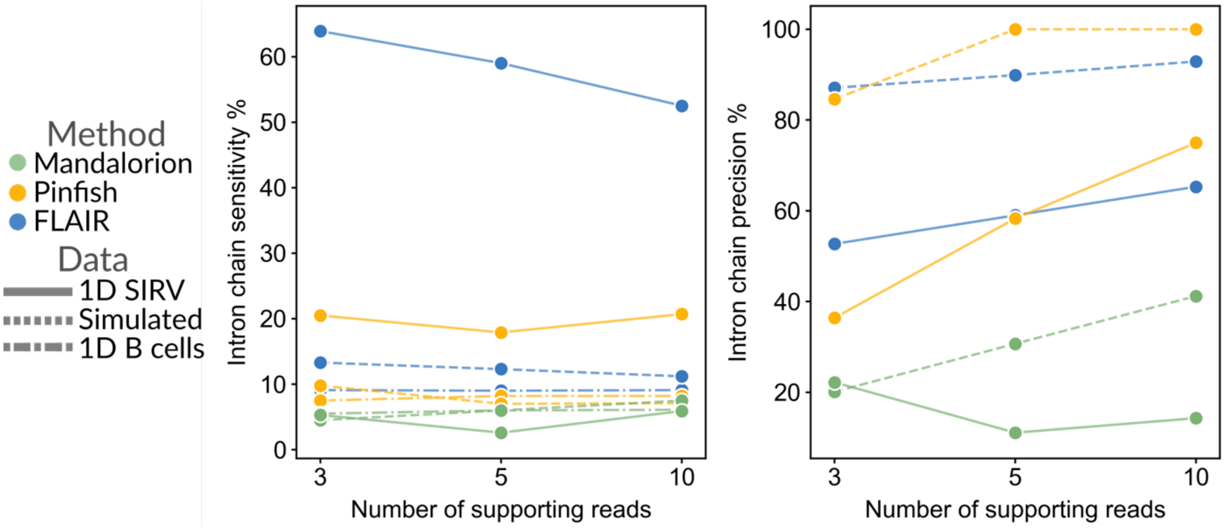

Supplementary Figure 5 | Intron chain level evaluation of software tools for nanopore isoform detection. Sensitivity (left) and precision (right) of isoforms using either Mandalorion (green), Pinfish (yellow), or Nanopore reads from either 1D sequencing of SIRVs (solid line), a simulated dataset (dashed line), or a subset of normal B cell 1D PromethION sequencing (dot-dash).

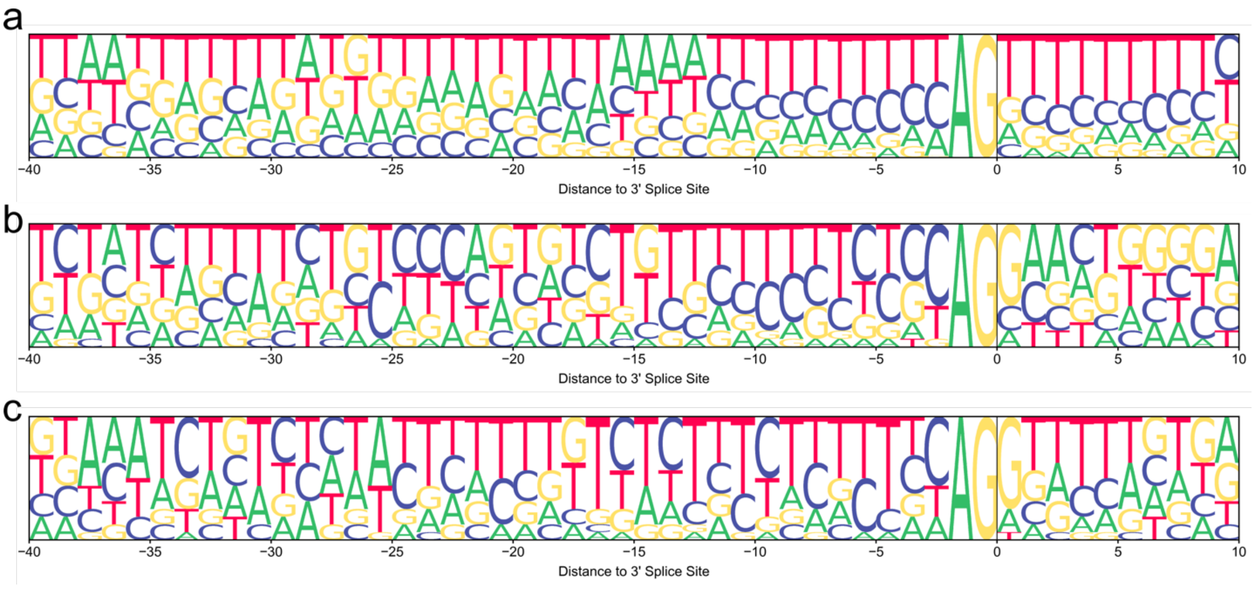

Supplementary Figure 6 | Splice site motifs identified from alternative splicing events between wildtype and mutant SF3B1. a, 3' splice site motif for alternative 3' splicing events identified in CLL short-read sequencing (n=65). **b,** 3' splice site motif using nanopore data for significant alternative 3' splicing events (n=15) with increased use of the proximal splice site. **c,** 3' splice site motif for significant IR events identified using nanopore data for the introns more spliced out in SF3B1 K700E (n=16)

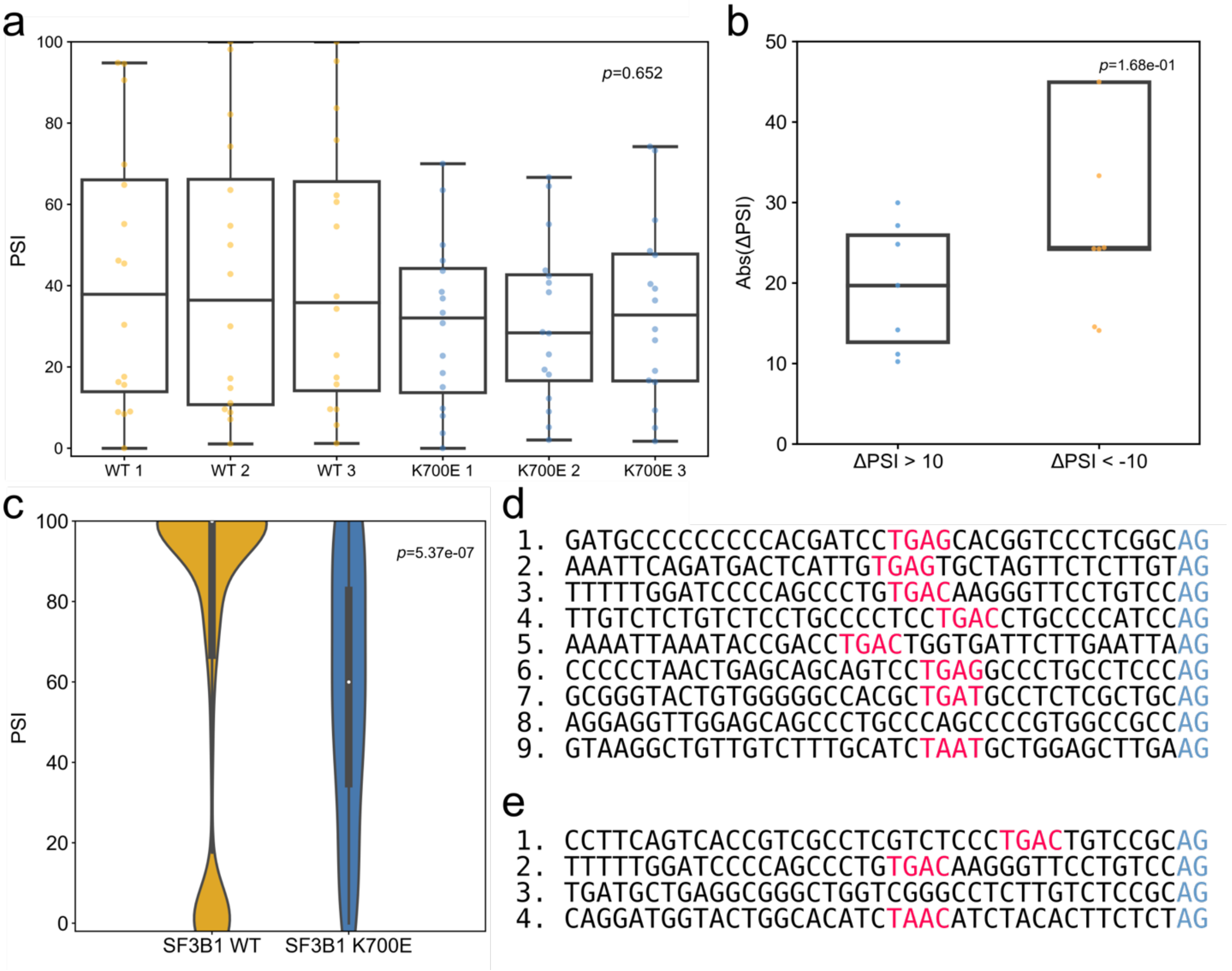

Supplementary Figure 7 | IR analysis of short read RNA-Seq data of Nalm-6 and TCGA BRCA samples with SF3B1 K700E. a, PSIs for 16 significant (corrected p < 0.1) IR events in 6 Nalm-6 samples, 3 with wildtype SF3B1 and 3 with SF3B1^{K700E}. The P-value is calculated from a Kruskal-Wallis H test. **b,** The change in PSI in significant intron retention events (corrected p < 0.1) identified in the Nalm-6 data that are more included in CLL SF3B1 K700E (blue) or more included in CLL SF3B1 WT (orange). Box-plots show median line, box limits are upper and lower quartile, and whiskers are 1.5x interquartile. **c,** 5 significant IR events were associated with SF3B1 K700E mutation in TCGA BRCA samples. The violin plots are made from individual PSIs for these IR events from: (SF3B1 WT) 801 samples without common splicing factor mutations and (SF3B1 K700E) 13 samples with SF3B1 K700E. Plot show median as white dot, box limits are upper and lower quartile, and filled area represents the entire range of the kernel density estimation. P-value is from a two-sided Mann-Whitney U test. **d,** 3' splice site sequences for the 9 significant IRs from the Nalm-6 analysis that were more included in the WT. Red: motifs that are similar to the branch point motif in Corvelo et al. 2010; yellow: 3' splice site AG dinucleotide. **e,** 3' splice site sequences for the 4 significant IR events that were more included in the WT identified in the TCGA BRCA samples.

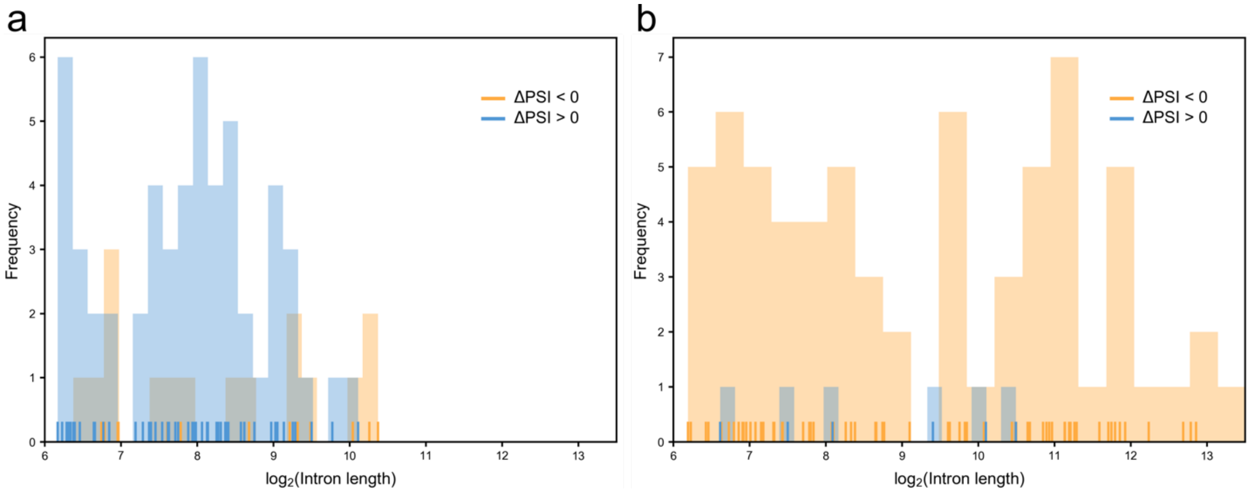

Supplementary Figure 8 | Nanopore and short-read retained intron lengths. a, Histogram of the lengths of significant intron retention (IR) events between *SF3B1*^{WT} and *SF3B1*^{K700E} identified in the long-read data. The ticks along the *x*-axis are the individual intron lengths. Orange, IR events more included in the wildtype. Blue, IR events more included in the mutant. **b,** Histogram of the lengths of significant IR events between mutant and wildtype *SF3B1* identified from short-read data. The coloring is the same as in (**a**).

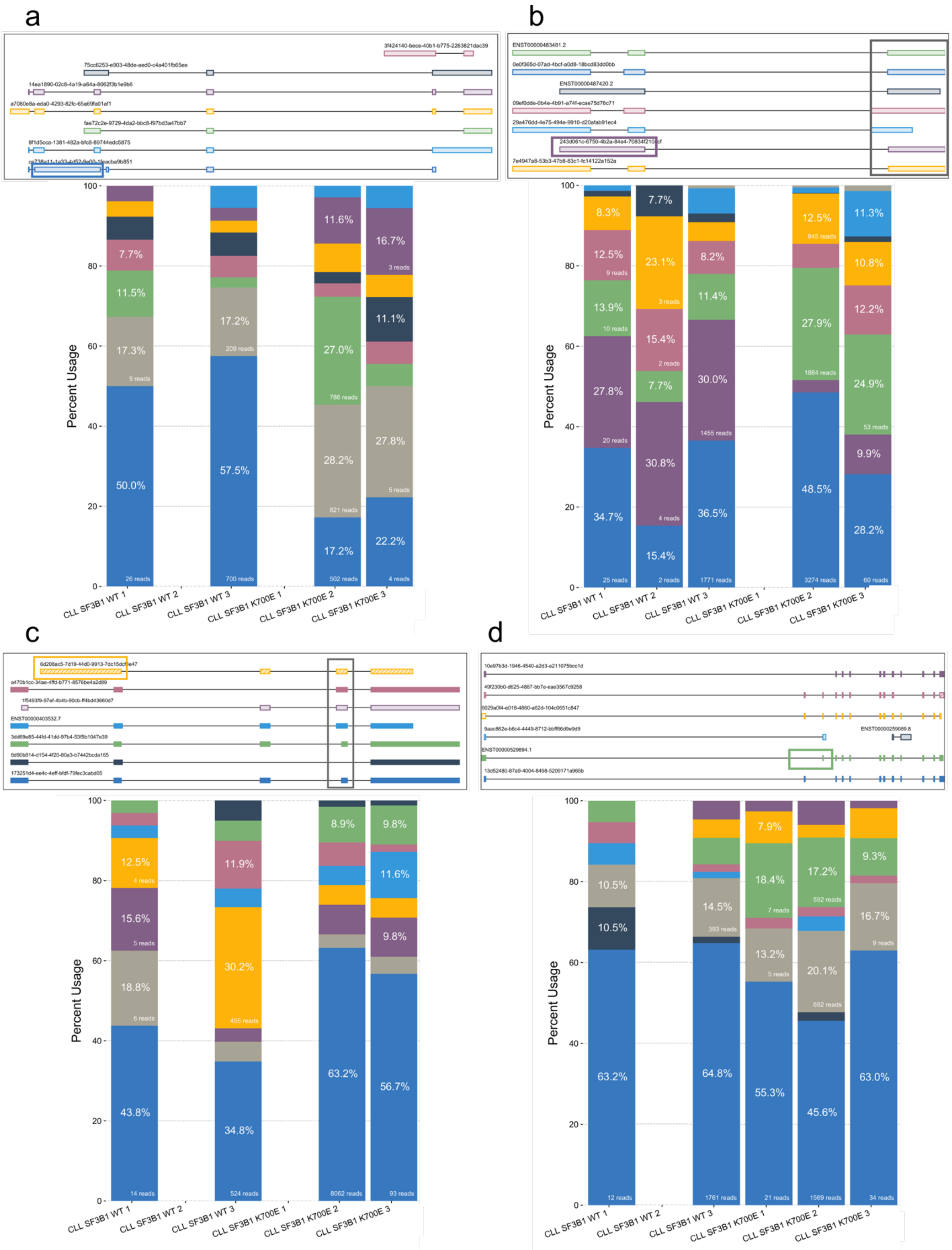

Supplementary Figure 9 | Examples of differential isoform usage. Isoform schematics and usage plots for isoforms that were differentially expressed in the nanopore SF3B1^{K700E} and SF3B1^{WT} data for the (**a**) LINC01089, (**b**) LINC01480, (**c**) XBP1, and (**d**) BLK genes. Isoforms with lower expression that are not included in the isoform schematic are represented as gray

bars. Samples with low coverage are not shown. Isoform fill scheme: solid indicates that the isoform contains an annotated start codon and a stop codon, hatched indicates that the isoform contains a premature termination codon and light-shading indicates that the isoform is missing an annotated start codon. Alternative 3’ SS are boxed in black.

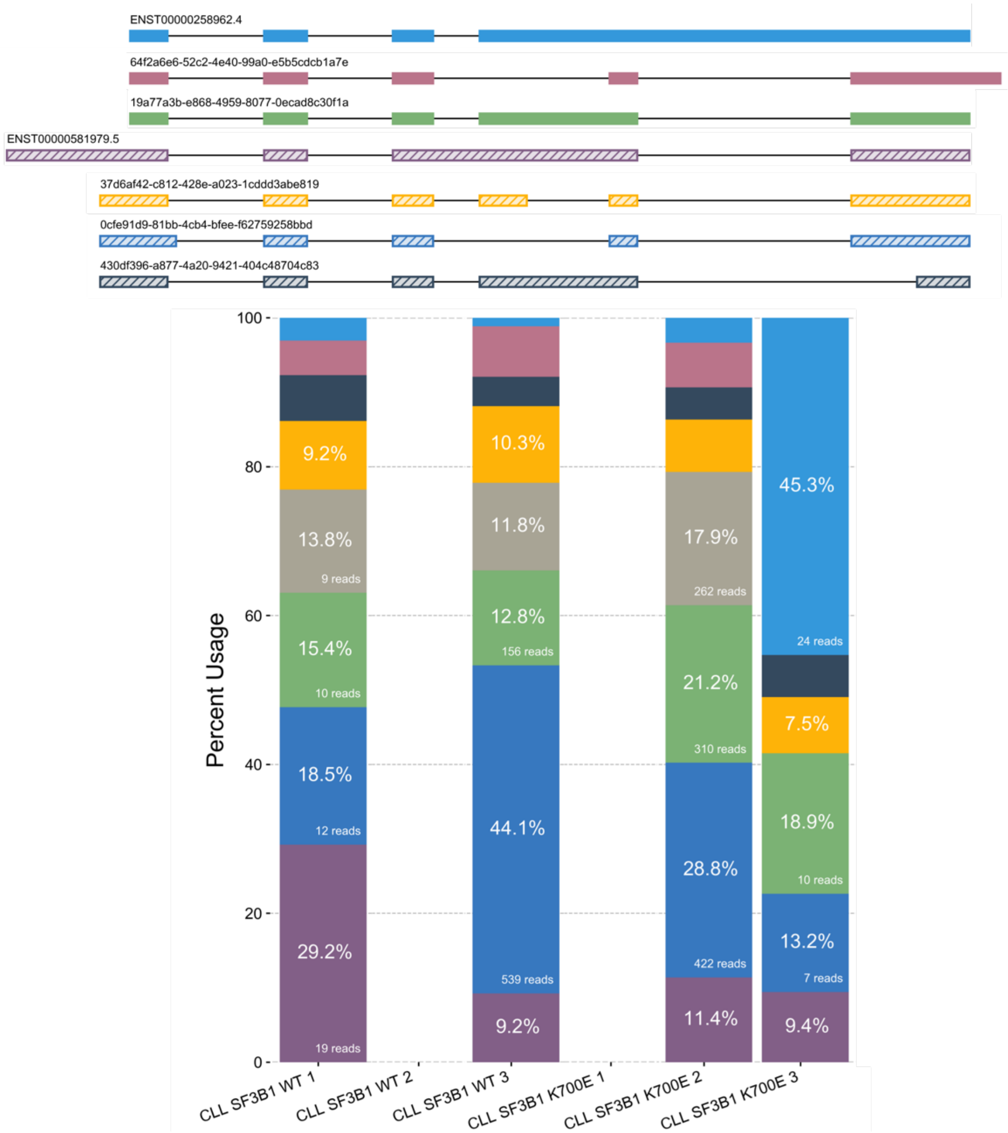

Supplementary Figure 10 | SRSF1 isoforms with known productivity. Top: Isoform structures, with exon fill denoting the predicted productivity (solid = productive, hatched = unproductive), Bottom: The percent usage corresponding in color to an isoform. All other minor isoforms not shown and instead represented in the gray usage bar. Samples with low counts are removed. The NMD-rule predicted productivity for the isoforms 37d6af42 (yellow, referred to as Isoform V in Sun et al. 2010), 19a77a3b (green), and ENST00000258962.4 (light blue) match the experimentally determined productivity determined in Sun et al. 2010. ENST00000581979.5 is detained in the nucleus and Isoform V is unproductive via degradation by NMD. The remaining isoforms were not included in previous productivity studies.

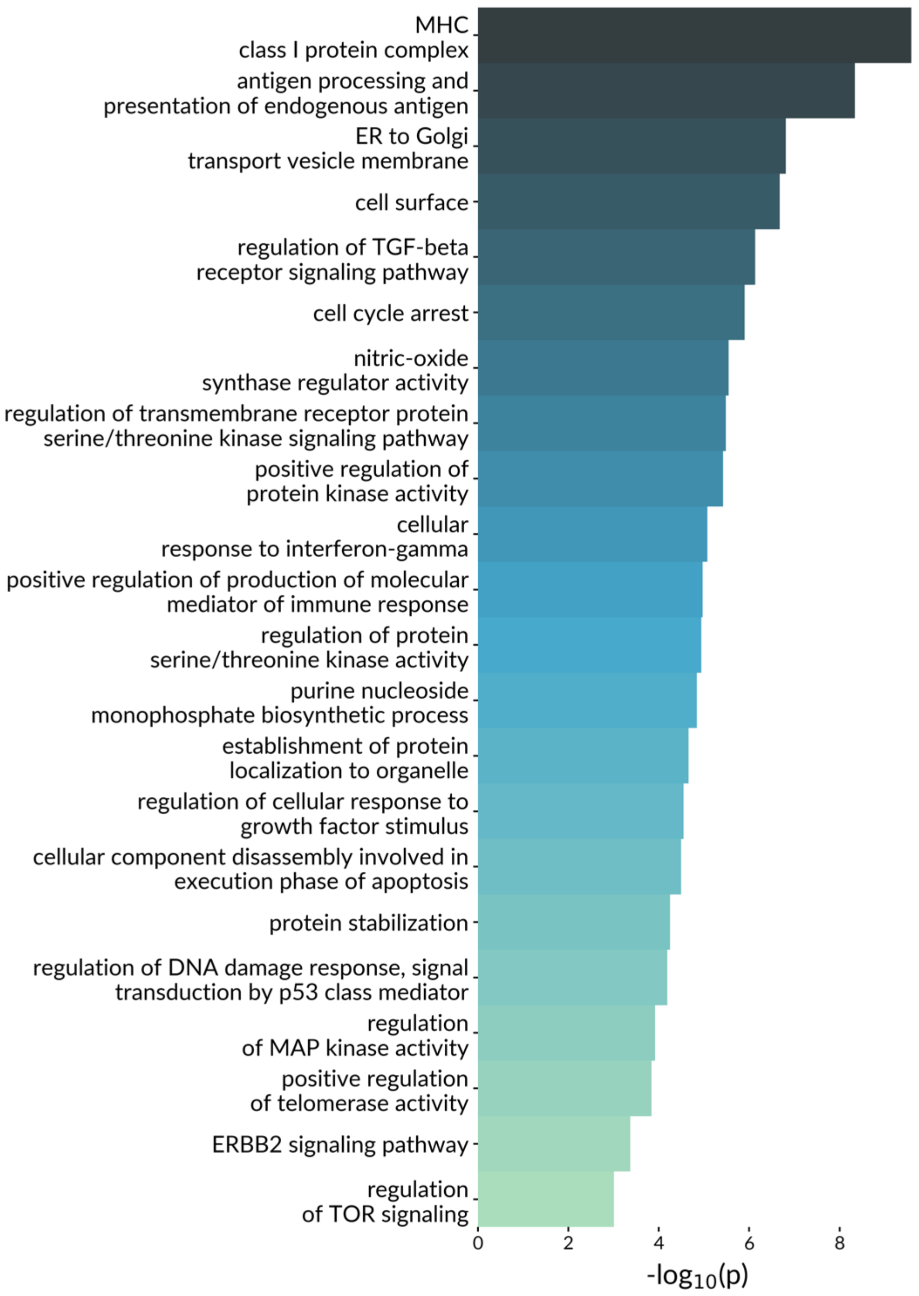

Supplementary Figure 11 | Top GO terms of genes with unproductive isoforms containing retained introns. Select GO term descriptions, with only the most significant overrepresented p-values calculated by GOseq displayed. GO terms with redundant keywords are reduced to the single most significant GO term. The unabridged list of GO terms is in Supplementary Table 1.

Supplementary Tables

Sample	cDNA starting concentration (ng/ul)
Promethion WT 1	3.78
Promethion WT 2	1.26
Promethion WT 3	3.42
Promethion MT 1	4.66
Promethion MT 2	8.58
Promethion MT 3	8.74
Promethion B cell 1	10.7
Promethion B cell 2	4.98
Promethion B cell 3	3.7

Supplementary Table 1: Starting cDNA concentrations prior to library preparation for PromethION samples. Concentration was measured using the Qubit dsDNA High Sensitivity kit.

Sample	Promethion sequencing batch	RNA batch
Promethion WT 1	2	1
Promethion WT 2	2	3
Promethion WT 3	1	1
Promethion MT 1	2	1
Promethion MT 2	1	1
Promethion MT 3	2	2
Promethion B cell 1	1	1
Promethion B cell 2	2	1
Promethion B cell 3	2	1

Supplementary Table 2: PromethION sample sequencing batches. Samples in the same sequencing batch had their libraries prepared at the same time and sequenced on the PromethION on the same day. RNA batch refers to the batch numbers according to those in Wang et al.[17], with the exception of WT 2, which is not included in that study. The sample IDs of the CLL *SF3B1*^{WT} samples are CW67 (WT 1) and CW95 (WT 3) from Wang et al.[17]; the IDs of the *SF3B1*^{K700E} samples are DFCI-5067 (MT 1), CLL043/CW109 (MT 2), and CLL032/CW84 (MT 3).
