# Supplementary material for: Full-length transcript characterization of SF3B1 mutation in chronic lymphocytic leukemia reveals downregulation of retained introns
Source: Nat Commun. 2020 Mar 18;11:1438. doi: 10.1038/s41467-020-15171-6 (PMC7080807; doi:10.1038/s41467-020-15171-6)
Supplement: Supplementary file 4 — Description of Additional Supplementary Files [file 41467_2020_15171_MOESM4_ESM.pdf]

Description of Additional Supplementary Files

File Name: Supplementary Data 1
Description: **FLAIR transcript models derived from nanopore sequencing data.** GTF file containing isoforms assembled from the FLAIR pipeline using all nanopore data generated from this study with a supporting read threshold of 3.

File Name: Supplementary Data 2
Description: **Isoform annotations in an extended PSL format for isoforms assembled from the FLAIR pipeline.** Each row is a FLAIR isoform. The first 21 columns follow PSL format, and columns after 21 contain additional information for each isoform. Spliced or intron retaining-isoforms are indicated with a 0 or 1 in column 22; productivity using the first start codon available in column 23; productivity using the longest ORF in column 24; the CLL SF3B1WT 1-3, CLL SF3B1K700E 1-3, and B cell samples 1-3 normalized isoform expression levels are in columns 25-33. The productivity categories productive, unproductive, no start codon, and no stop codon are indicated with PRO, PTC, NGO, and NST respectively.

File Name: Supplementary Data 3
Description: **Alternative splicing events identified using nanopore sequencing data.** The alternative splicing events identified by FLAIR diffSplice and DRIMSeq that had p-values < 0.1 and abs(dPSI) > 10.

File Name: Supplementary Data 4
Description: Differentially used isoforms identified using nanopore sequencing data. Differen tially used isoforms as identified using FLAIR diffExp with p-value < 0.1 and abs(dPSI) > 10, co rrecting for batches as specified in Supplementary Table 2.

File Name: Supplementary Data 5
Description: **GO enrichment of genes with intron retention downregulated in SF3B1K700E relative to SF3B1WT determined by nanopore sequencing data.** GOseq results, with only GO terms with overrepresented p-values < 0.05 included.
